# A new candidate vaccine for human brucellosis based on influenza viral vectors: a preliminary investigation for the development of an immunization schedule in a guinea pig model

**DOI:** 10.1186/s40249-021-00801-y

**Published:** 2021-02-16

**Authors:** Dina Bugybayeva, Zhailaubay Kydyrbayev, Nadezhda Zinina, Nurika Assanzhanova, Bolat Yespembetov, Yerken Kozhamkulov, Kunsulu Zakarya, Sholpan Ryskeldinova, Kaissar Tabynov

**Affiliations:** 1 Research Institute for Biological Safety Problems, 15 Momushuly, Gvardeyskiy, 080409 Kazakhstan; 2 Kazakh National Agrarian University, 8 Abay Avenue, Almaty, 050010 Kazakhstan; 3 Research Institute of Cardiology and Internal Medicine, 120 Aiteke bi, Almaty, 050000 Kazakhstan

**Keywords:** Human brucellosis, Influenza viral vectors, Vaccine candidate, Protection, Guinea pigs, Immunization route, Vaccination dose

## Abstract

**Background:**

A new candidate vector vaccine against human brucellosis based on recombinant influenza viral vectors (rIVV) subtypes H5N1 expressing *Brucella* outer membrane protein (Omp) 16, L7/L12, Omp19 or Cu–Zn SOD proteins has been developed. This paper presents the results of the study of protection of the vaccine using on guinea pigs, including various options of administering, dose and frequency. Provided data of the novel vaccine candidate will contribute to its further movement into the preclinical stage study.

**Methods:**

General states of guinea pigs was assessed based on behavior and dynamics of a guinea pig weight-gain test. The effectiveness of the new anti-brucellosis vector vaccine was determined by studying its protective effect after conjunctival, intranasal and sublingual administration in doses 10^5^ EID_50_, 10^6^ EID_50_ and 10^7^ EID_50_ during prime and boost vaccinations of animals, followed by challenge with a virulent strain of *B. melitensis* 16 M infection. For sake of comparison, the commercial *B. melitensis* Rev.1 vaccine was used as a control. The protective properties of vaccines were assessed by quantitation of *Brucella* colonization in organs and tissues of infected animals and compared to the control groups.

**Results:**

It was observed a gradual increase in body weight of guinea pigs after prime and booster immunization with the vaccine using conjunctival, intranasal and sublingual routes of administration, as well as after using various doses of vaccine. The most optimal way of using the vaccine has been established: double intranasal immunization of guinea pigs at a dose of 10^6^ EID_50_, which provides 80% protection of guinea pigs from *B. melitensis* 16 M infection (*P* < 0.05), which is comparable to the results of the effectiveness of the commercial *B. melitensis* Rev.1 vaccine.

**Conclusions:**

We developed effective human vaccine candidate against brucellosis and developed its immunization protocol in guinea pig model. We believe that because of these studies, the proposed vaccine has achieved the best level of protection, which in turn provides a basis for its further promotion.

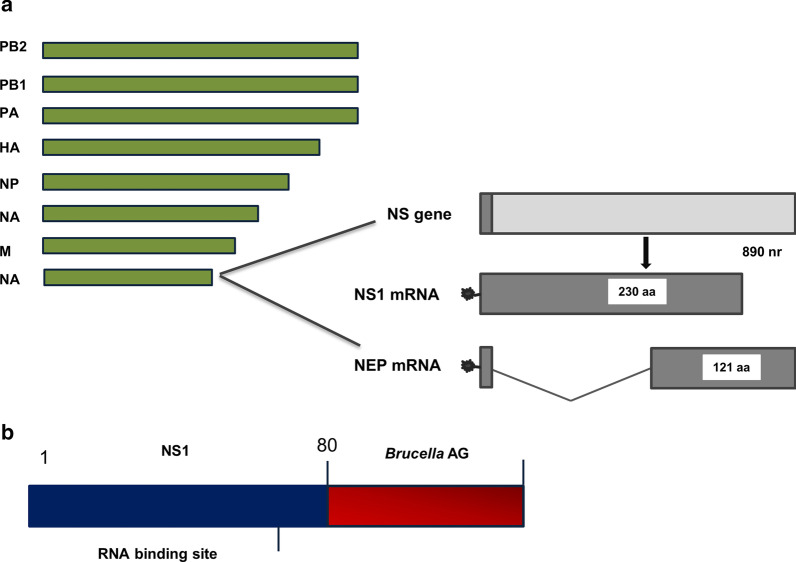

## Background

Brucellosis is a chronic infectious disease of humans and animals, which is included in the list of quarantine diseases as a social threat factor. There are ten known species of the causative agent of brucellosis, which includes those that pose a threat to human health—*Brucella melitensis*, *B. abortus*, *B. suis* and *B. canis* [1, 2]. *B. melitensis* that causes the most severe and acute form of infection is responsible for 80–90% of human brucellosis cases [3]. Most cases of brucellosis in humans are the results of occupational exposure to the bacteria and consumption of infected dairy products [4].

Despite the fact that brucellosis is amenable to antibiotic therapy, it seriously weakens the human body and many patients require long-term recovery. In addition, due to the intracellular tropism of *Brucella*, only a very limited number of antibiotics can be used to treat this infection [5]. It is important to note that in 5–40% of cases the antibiotic therapy results in relapses of the disease, which requires protracted treatment using different combinations of antibiotics [6]. This fact, as well as the lack of a licensed anti-brucellosis vaccine for humans represent a serious problem for the brucellosis endemic areas.

Earlier, USSR and China were widely using live attenuated vaccines based on *B. abortus* 19 BA or *B. melitensis* 104 M strains for human vaccination [7]. Normally, in vaccinated people, these vaccines ensured a short-term immune response and were also accompanied by high reactogenicity and hypersensitivity, especially when repeated doses of the vaccine were administered.

One of the most important strategies in the development of safe and effective anti-brucellosis vaccines is the use of live genetically modified vectors—non-pathogenic microorganisms (bacteria and viruses) that produce brucellosis antigens. Nowadays, *Lactococcus Lactis* [8], *Escherichia coli* [9], *Salmonela enterica* [10] and *Semliki Forest virus* (SFV) [11] are used as vectors for the expression of brucellosis proteins in vivo. It has been proved experimentally that the tested bacterial (intracellular) and viral vectors are capable of infecting a wide range of cell types and expressing brucellosis antigens within the infected cell.

Previously, we used recombinant Influenza A viral vectors of both subtypes a H5N1 for prime vaccination and H1N1 for booster vaccination that express brucellosis immunodominant outer membrane proteins (Omp) 16 and ribosomal L7/L12 in order to develop a new *Brucella abortus* vaccine (Flu-BA) against brucellosis in cattle [12, 13]. Now, the Flu-BA vaccine is registered in Kazakhstan (registration certificate #RK-VP-1-3775-19 dated from January 14, 2019) and is at the stage of commercialization for vaccination of cattle against *B. abortus* infection.

The effectiveness of cattle vaccination is comparable to the results of using the commercial *B. abortus* S19 vaccine. The use of influenza viral vectors (IVV) subtype H5N1 may serve as an additional factor in increasing the effectiveness of the vaccine for humans. The fact is that there is no pre-existing immunity to influenza virus H5N1 in human population. Therefore, IVV of the H5N1 subtype has more opportunities for replication and expression of brucellosis proteins.

In this study, we used the entire existing stocks of previously obtained recombinant IVV type A of subtype H5N1 that express *Brucella* Omp 16 and 19, ribosomal L7/L12 or Cu–Zn superoxide dismutase (SOD) from the open reading frame of the NS1 gene at amino acid position 80 [14].

Brucella antigens Omp 16 and 19, L7/L12 and SOD induce a pronounced cellular response which is necessary for protection against brucellosis infection [15–17]. It should be noted that the proteins expressed by IVV are immunodominant and common (genetically similar by 95–99%) for *B. melitensis*, *B. abortus*, *B. suis*, and *B. canis* [18–20]. In this regard, we have developed an anti-brucellosis tetravalent IVV based human vaccine candidate expressing the brucellosis proteins Omp16, L7/L12, Omp19 and SOD.

This article presents the results of studying clinical observation of general states of guinea pigs and protectiveness of the anti-brucellosis human vaccine with different modes of administration, with different doses and frequencies of vaccination in guinea pigs.

## Methods

### Generation of viral constructs and preparation of vaccine samples

**Fig. 1:**
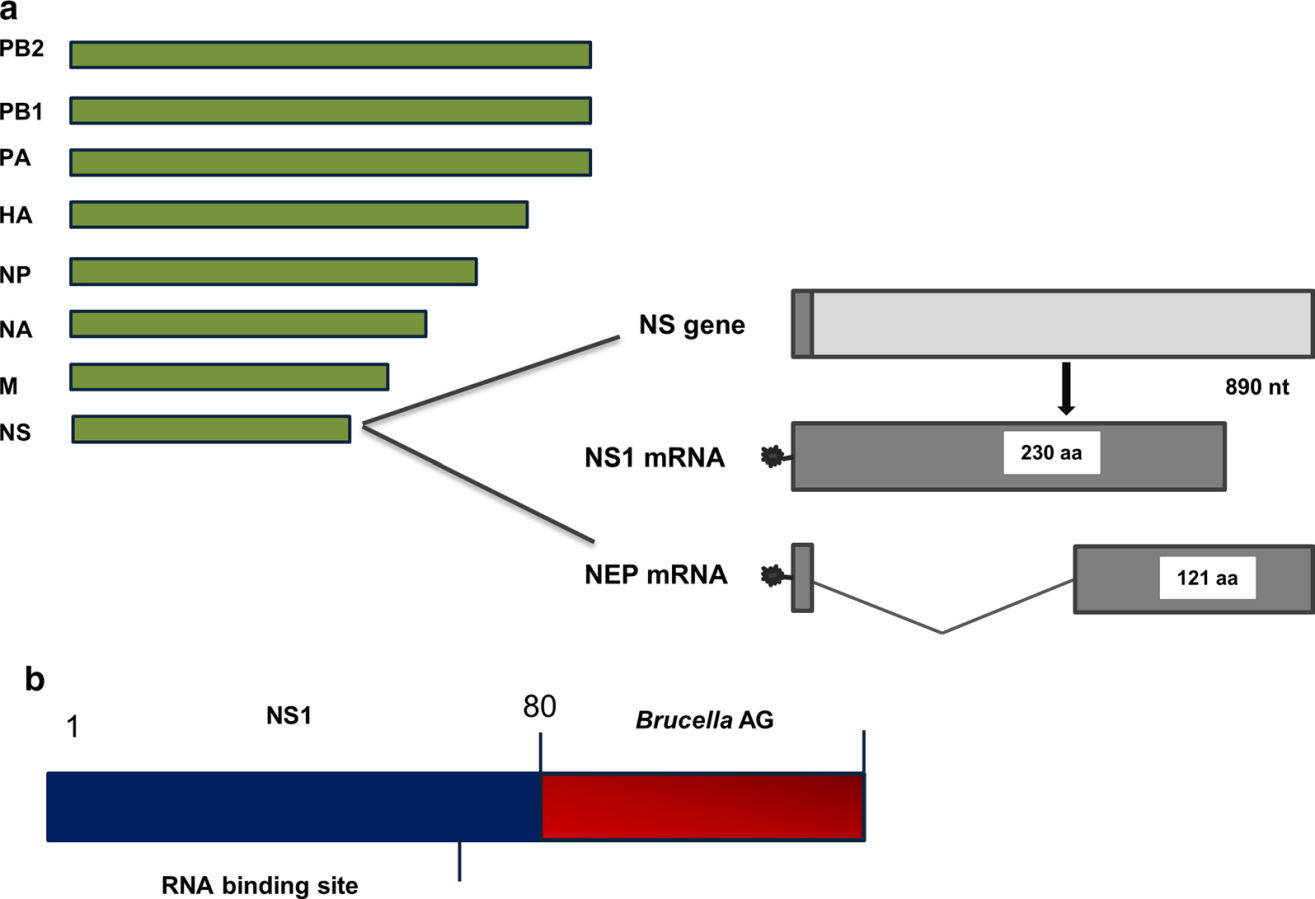
Schematic representation of the recombinant influenza viral vector construction destined to generate a vaccine against brucellosis. **a** Full size NS1 protein virulence factor for antagonizing interferon system and **b** deletion part of NS1 replaced by *Brucella* immunodominant proteins NS1-Omp 16, NS1-Omp 19, NS1-L7/L12 and NS1-Cu–Zn SOD. The blue box and red box **b** represent RNA binding site of NS1 and part of NS1 protein at amino acid position 80 for insertion brucellosis segment, respectively. PB2, PB1 and PA: Influenza A virus RNA polymerase subunits; HA: Hemagglutinin; NP: Nucleoprotein; NA: Neuraminidase; M: Matrix; NS1: Non–structural protein 1; NEP: Nuclear export protein. The scheme is not drawn according to scale

Influenza viruses were obtained by a standard reverse genetics method using eight bi-directional plasmids pHW2000. In this study, we used IVV type A of H5N1 subtype that express *Brucella* proteins Omp16, Omp19, L7/L12 or SOD from NS1 open reading frames at amino acid position 80. A detailed procedure for the construction of the IVV has been described previously [14]. Schematic picture for IVV is presented in Fig. 1. Obtained recombinant influenza vectors (Flu-NS1-80-Omp16, Flu-NS1-80-L7/L12, Flu-NS1-80-Omp19, Flu-NS1-80-Cu–Zn SOD) were then used to produce vaccine for immunization guinea pigs. Briefly, the viral vector was accumulated in ten-day old chicken embryos (CE) at 34 °C for 48 h. The IVV titer was determined by hemagglutination assay based on the generally accepted method as reported previously [21].

Allantoic suspensions of IVV were clarified, purified and concentrated by ultracentrifugation and then the resulting material was sent to diafiltration and sterilizing filtration and then combined with a sterile stabilizing medium containing 12% soy peptone (Sigma-Aldrich, USA) and 6% sucrose (Sigma-Aldrich, USA). The combined material was added to 1 ml ampoule, lyophilized and stored at 2–8 °C. The lyophilized vaccine was resuspended in phosphate buffered saline (PBS) prior to vaccination of the animals.

### Bacterial strains

The virulent *B. melitensis* 16 M strain was obtained from the collection of microorganisms of the Research Institute for Biological Safety Problems (RIBSP). Bacterial cells were cultured under aerobic conditions on a Brucella Agar Base nutrient medium (Sigma-Aldrich, USA) at 37 °C. All experiments with live *Brucella* cells were conducted in Biosafety level 3 facility (BSL-3) at the RIBSP. Infected animals were contained in specialized animal BSL-3 facility.

### Bioethics and groups of experimental animals

The studies were conducted in accordance with national and international regulations and guidelines for the handling and use of laboratory animals. The study protocol was approved by the RIBSP Bioethics Committee (Protocol no. 0418/04). The animals contained in cages on 12 light/12 dark cycle and were fed ad libitum with standard rodent diet and had no water restrictions. Animals from control and experimental groups were kept isolated from each other throughout the whole experiment. This study used conventional bred female guinea pigs weighing 250–330 g (National Center for Expertise of Drugs, Medical Products and Equipment, Kazakhstan).

### Immunization of guinea pigs

To determine the method of applying the vaccine, five groups (*n* = 5 animals per group) were formed, that is, three experimental and two control groups. The experimental groups were administered with vaccine conjunctivally (*c.*) in a volume of 100 μl (50 μl in each eye) or intranasally (*i.n.*) in a volume of 200 μl (100 μl in each nasal cavity) or sublingually (*s.l.*) in a volume of 200 μl. The infectious titer of the IVV in the experimental vaccine samples was 6.14–6.75 log10 EID_50_/animal. On day 21 after the prime vaccination, guinea pigs were boosted according to the immunization protocol. The animals in negative control group were injected subcutaneously (*s.c.*) with 200 μl of PBS. In the positive control group, a dose of 10^5^ CFU/animal commercial *B. melitensis* Rev.1 vaccine (Antigen LLP, Kazakhstan) was injected once *s.c.*.

Further on, to determine the protective dose and the frequency of immunization, the vaccine was administered *i.n*. Three doses of the vaccine—10^5^ EID_50_, 10^6^ EID_50_ and 10^7^ EID_50_ were evaluated in six experimental groups of guinea pigs (*n* = 5 animals per group), including control groups, after prime-boost immunization according to the protocol presented above.

In order to assess weight changes associated with the vaccine, immunized guinea pigs were clinically monitored with weekly weighing for 42 days upon both prime and boost vaccinations. The clinical assessment was evaluated based on parameters including the survival rate, general condition, behavior and dynamics of animals’ weight.

### Assessment protectiveness of the vaccine

On the day 21 after the boost immunization, the guinea pigs (45 animals in total) from the vaccinated and control groups were *s.c.* infected with a virulent strain of *B. melitensis* 16 M infection at a dose of 20 CFU/animal. Guinea pigs from positive (*n* = 10) control groups were infected in a similar way on day 42 after a single vaccination with *B. melitensis* Rev.1. Thirty days after infection, all guinea pigs were euthanized with CO_2_ and lymph nodes (retropharyngeal, lower cervical and right and left inguinal), liver, spleen and bone marrow were extracted aseptically. Bacteriological examination and evaluation of the results were carried out according to the previously described method [23]. Briefly, after tenfold serial dilution the tissue homogenates in 0.1% Triton X-100-PBS solution were inoculated into plates with *Brucella* Base Agar (HiMedia, India) and incubated at 37 °C for two weeks with periodical counting of the bacterial colonies during this period.

The concentration of bacteria (colony forming units (CFU)/g per tissue) in tissue samples was determined by standard colony counting. An animal was considered infected if a *Brucella* colony was found in culture of one or more organs. The results of the bacteriological study were assessed according to several parameters: (a) vaccination efficacy or the number of animals (expressed in %) from which no *Brucella* colonies were isolated in any animal samples; (b) generalization of the infectious process or the index of infection (the number of organs and lymph nodes of animals from which *Brucella* was isolated and which is represented in the arithmetic mean); (c) the intensity/severity of the infectious process or the degree of *Brucella* colonization of organs and lymph nodes (LN) expressed in log_10_ CFU/g of tissue.

### Statistical analysis

The index of infection and *Brucella* colonization in tissues between groups were analyzed with one- way or two-way ANOVA followed by multiple comparisons of the Turkey test, Sidak or Dunnett’s tests. The variance in protective efficacy of animal groups was compared by one-sided Fisher exact test. *P* < 0.05 was considered significant. Means are reported with standard errors (SEM). Statistical analysis of all experimental data was performed using Graph Pad Prizm Software Version 8.0 (Graph Pad Software Inc., La Jolla, CA, USA). The experiments have been repeated, and the results were reproducible.

## Results

### Assessing general states of the guinea pigs after vaccination

The effect of the vaccine on guinea pigs was evaluated with different ways of administering, with different doses and frequencies of the use of vaccine in comparison with a negative (PBS) control group.

It was found that the vaccine was safe in guinea pigs in different ways of vaccine administering—*c.*, *i.n.* and *s.l.*, as well as in various doses in primary and secondary immunization (prime and boost). There were no animal deaths or signs of disease by the end of the observation period. In general, the condition of the animals, both in the control and in the experimental groups, was satisfactory in terms of physical activity, appetite and general health condition.

**Fig. 2:**
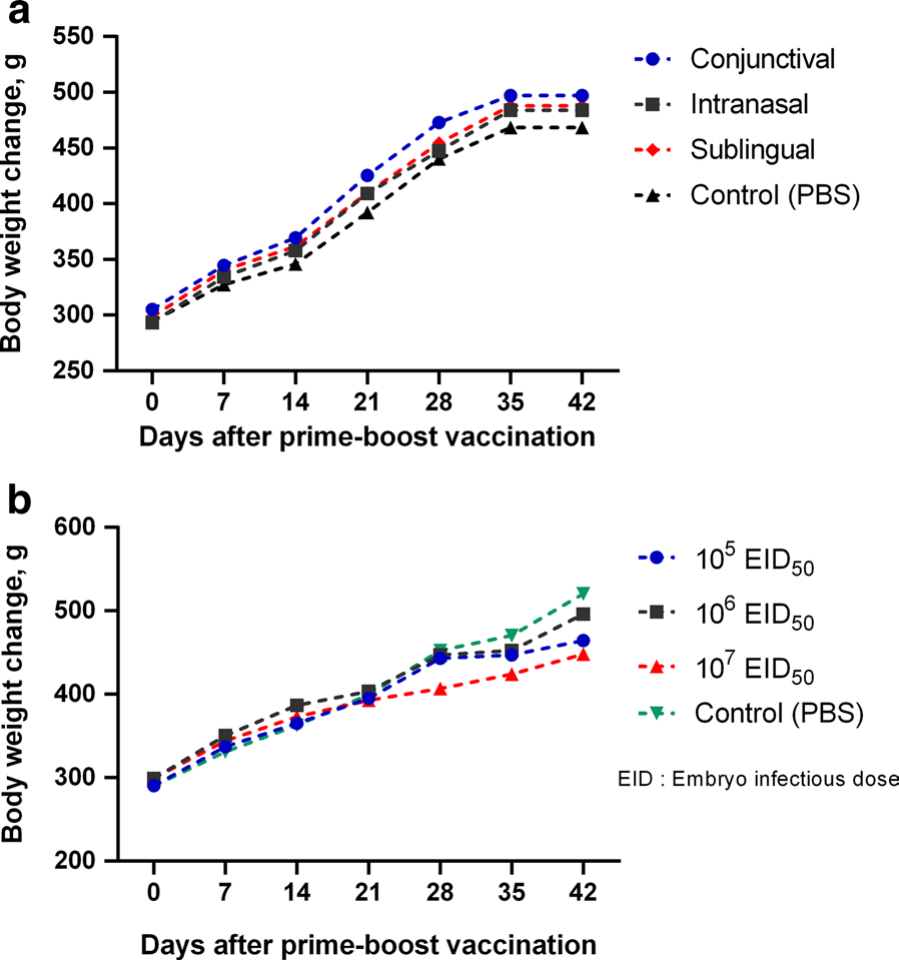
Dynamics of body weight change of guinea pigs on day 42 after prime-boost immunization. Animals were immunized with vaccine candidate against human brucellosis at different routes of immunization (**a**) and various doses (**b**). Statistical analysis was performed with two-way ANOVA followed by Dunnett’s multiple comparisons test showed that during 42-day body weight measurement between the PBS control and vaccinated groups were not significant. *P* < 0.05 values were considered significant

Evaluation of changes in animal body weight within 21 days after prime-boost immunization showed that the weight of animals (on average) increased in all test groups (Fig. 2) by 45–58% and amounted to 158–175 g which was comparable to the control group—55% and 162 g.

No group was differed significantly in mean of animal weight from the negative (PBS) control group.

### Protectiveness of viral vector based vaccine candidate at different ways of its administration against *B. melitensis* 16 M infection

The protective efficacy of the vaccine candidate was evaluated in guinea pigs using *c.*, *i.n.* and *s.l.* routes of administration and compared with reference *B. melitensis* Rev.1 vaccine or PBS control groups.

The protective efficacy of the vaccine was assessed by parameters as the index of infection, the efficacy of vaccination and the number of bacteria of the virulent strain of *B. melitensis* 16 M obtained from organs and tissues of vaccinated and unvaccinated animals.

The results of the bacteriological study showed that with various mucosal routs of immunization, the new anti-brucellosis vaccine candidate provided protection at a level of 1.64 to 2.30 log_10_ units. In comparison with the unvaccinated control group (PBS), all vaccine samples, regardless of the route of administration, ensured protection of guinea pigs from *B. melitensis* 16 M infection (α = 0.0001–0.02) (Table 1).

**Table 1:** Degree of protective efficacy of the vaccines by different routes of administration in guinea pigs

Vaccine	Route of administration	Log_10_ CFU/animal^a^ (mean ± SE)	Log_10_ protection ^b^	Value (*P*) ^c^
Influenza viral vector based brucellosis vaccine candidate	*c*	0.56 ± 0.22	2.3	< 0.003
	*i.n*	0.06 ± 0.04	2.8	< 0.0001
	*s.l*	1.22 ± 0.29	1.64	< 0.02
Commercial vaccine *B. melitensis* Rev.1	*s.c*	0.48 ± 0.18	0.38	< 0.0005
Control (PBS)	*i.n*	2.86 ± 0.19	0.00	-

^a^Degree of protective efficacy of the vaccines was evaluated by the isolation rate of *Brucella* from organs and tissues of guinea pigs challenged with the virulent strain of *B. melitensis* 16 M infection

^b^Log_10_ protection units were obtained by subtracting the mean log_10_ CFU of the control (PBS) group from the mean of log_10_ CFU for the experimental group

^c^Compared with control group (PBS)

*CFU* colony forming units, *PBS* phosphate-buffered saline, *c.* conjunctivally, *i.n.* intranasally, *s.l.* sublingually

Protective efficacy of vaccines as evaluated by the isolation rate of *Brucella* from the tissues of control and experimental groups of guinea pigs on day 30 after challenging with the virulent strain of *B. melitensis* 16 M. Animals were vaccinated with the vector vaccine by prime-boost *c.*, *i.n.*, *s.l.* at interval of 21 days, and with *B. melitensis* Rev.1 by single *s.c.* vaccination. Guinea pigs in negative control group were injected with PBS. The challenge of animals was performed with *B. melitensis* 16 M at a dose of 1.3 log_10_ CFU/animal using *s.c.* route.Statistical analysis was performed using two-way ANOVA followed by Tukey’s multiple comparisons test

Significant protection of the vaccine was observed after *i.n.* administration (2.8 log_10_), whereas for *s.l.* immunization, the unit of protection was 1.64 log_10_. In case when the vaccine administered by *c.* route the unit of its protection was 2.3 log_10_, which was comparable to the commercial *B. melitensis* Rev.1 vaccine results.

**Fig. 3:**
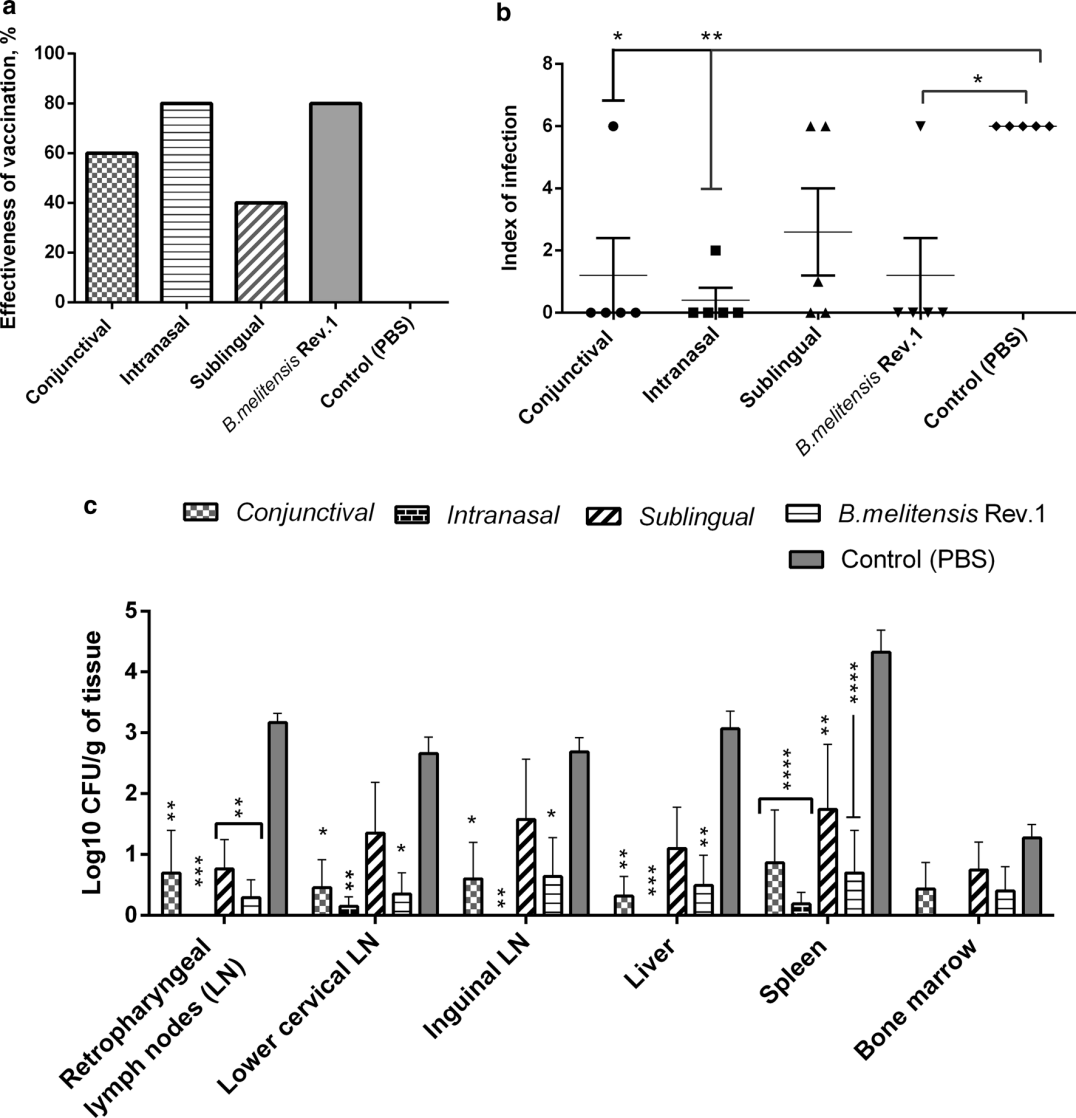
Protective efficacy of the vaccines in guinea pigs when administered by different routes. Protective efficacy of vaccines as evaluated by the effectiveness of vaccination (**a**), index of infection (**b**) and isolation rate of *Brucella* (**c**) from the tissues of control and experimental groups of guinea pigs on day 30 after challenging with the virulent strain of *B. melitensis* 16 M. Animals were vaccinated with the vector vaccine by prime-boost conjunctival (*c.*), intranasal (*i.n.*), sublingual (*s.l.*) at interval of 21 days, and with *B. melitensis* Rev.1 by single subcutaneous (*s.c.*) vaccination. Guinea pigs in negative control group were injected with PBS. The challenge of animals was performed with *B. melitensis* 16 M at a dose of 20 CFU/animal using *s.c.* route. Bacteriological evaluation was assessed by the index of infection in animals (the arithmetic mean ± standard error was given for each group; the number of organs and lymph nodes from which *Brucella* was isolated for each animal) and by counting *Brucella* colonies in tissues, where data is expressed as log_10_ CFU/g. Statistical difference between groups was indicated with asterisks and statistical analysis for (B) was performed using a one-way ANOVA followed by Dunnett's multiple comparisons test and (*, *P* < 0.01; **, *P* < 0.002) and for **c** two-way ANOVA, Tukey’s multiple comparisons test (*P = 0.04–0.01; **P = 0.009–0.001; ***P = 0.0004–0.0002, ****P < 0.0001)

According to the index of infection (Fig. 3b), significant protection compared to the control group (PBS) was achieved in the groups vaccinated *c.* (*P* < 0.01; vaccination efficiency 60%) and *i.n.* (*P* < 0.002, vaccination efficiency 80%), as well as in animals vaccinated with *B. melitensis* Rev.1 (*P* < 0.005; vaccination efficiency 80%). It should be noted that the index of infection in *i.n.* vaccinated animals was similar (*P* > 0.99) to the index in animals immunized with the commercial *B. melitensis* Rev.1 vaccine.

Based on the results of these studies, the *i.n.* route of vaccine administration was applied for further research in order to determine the optimal dose for immunization.

### Study of protective efficacy of a novel vaccine candidate at different doses and after infection with *B. melitensis*

In this study, the protectiveness of the vaccine was assessed at various doses of *i.n.* vaccine administration after prime-boost immunization, as well as after challenge of guinea pigs with the virulent *B. melitensis* 16 M strain.

The evaluation of the protective efficacy of the tetravalent vector brucellosis vaccine against the infection was carried out according to the following parameters (1) determination of the effectiveness of vaccination (the degree of complete protection against infection, expressed in percent), (2) study of the infection index, as well as by counting *Brucella* colonies in the tissues and lymph nodes of guinea pigs 30 days after infection.

Bacteriological studies of the organs and lymph nodes of infected animals showed that the tested doses of the recombinant vector vaccine, as well as the commercial vaccine, provided significant protection (*P* < 0.04 versus the unvaccinated group) of guinea pigs against *B. melitensis* 16 M infection ranging from 1.64 to 2.8 log_10_ units. Our results showed that the highest level of protection (vaccination efficiency) against infection in guinea pigs was in the groups immunized at doses of 10^6^ EID_50_ and 10^7^ EID_50_ (80%) compared with the control group (PBS) after the challenge, in which the infection rate was 100% and these data were statistically significant (*P* < 0.04). It should be noted that in the group vaccinated with *B. melitensis* Rev.1 the vaccination efficiency was also 80% (Table 2).

**Table 2:** Rates of protection in guinea pigs after challenge with the virulent strain *B. melitensis* 16 M

Immunization group (prime-boost)	Total animals	Isolation of *B. melitensis* in animals, *n* (%)	Value (*P*)^a^	
			( +) control^b^	(−) control^c^
Vector vaccine at dose of 10^5^ EID_50_	5	2 (40)	> 0.05	> 0.05
Vector vaccine at dose of 10^6^ EID_50_	5	1 (20)	> 0.05	< 0.05
Vector vaccine at dose of 10^7^ EID_50_	5	1 (20)	> 0.05	< 0.05
*B. melitensis* Rev.1	5	1 (20)	-	< 0.05
Control (PBS)	5	5 (100)	< 0.05	-

^a^In comparison with control untreated PBS or *B. melitensis* Rev.1 groups

^b^Animals immunized with *B. melitensis* Rev.1 vaccine

^c^Animals inoculated with PBS. *EID*_*50*_ 50 percent embryo infectious dose

Guinea pigs were immunized twice *intranasally* 21 days apart with a new vaccine candidate at dose 10^5^ EID_50_, 10^6^ EID_50_ and 10^7^ EID_50_ or a single delivery of the vaccine *B. melitensis* Rev.1. via *s.c.* immunization. Animals challenged with virulent strain of *B. melitensis* 16 M at a dose of 1.3 log_10_ CFU/animal using *subcutaneous* route. Statistical analysis was performed using a one-sided Fisher’s exact test. *P*-value less than 0.05 (< 0.05), *P*-value higher than 0.05 (> 0.05)

**Fig. 4:**
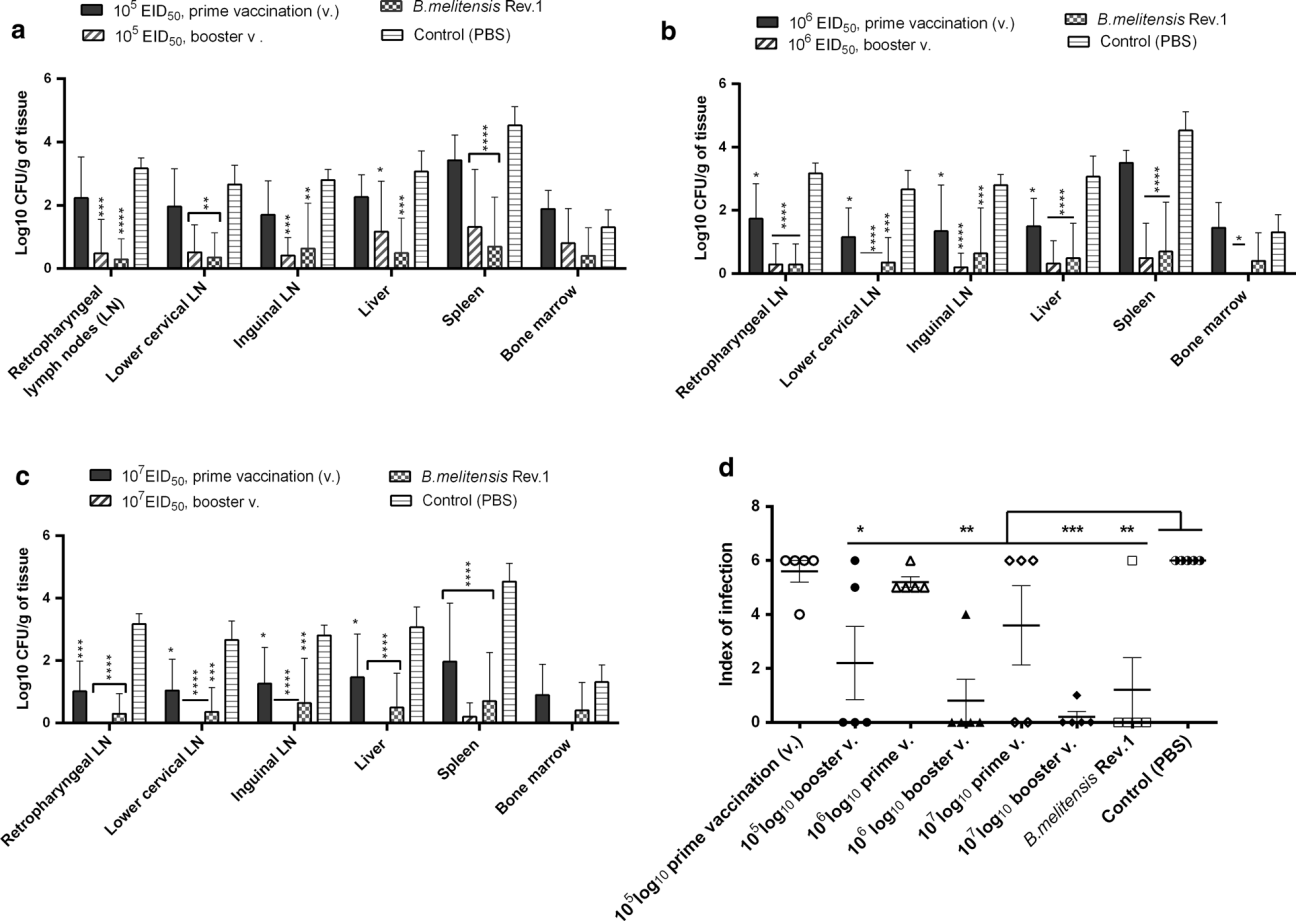
Protectiveness of vaccine samples at different doses in guinea pigs. **a–c** Colonization of *B. melitensis* in organs and tissues of prime-boost vaccinated guinea pigs and **c** index of *Brucella* infection upon challenge with *B. melitensis* 16 M. Guinea pigs were immunized twice *i.n.* 21 days apart with a new vaccine candidate at dose 10^5^ EID_50_, 10^6^ EID_50_ and 10^7^ EID_50_ or a single delivery of commercial vaccine *B. melitensis* Rev.1. via *s.c.* immunization. Guinea pigs of positive control groups were injected with PBS. Animals challenged with virulent strain of *B. melitensis* 16 M at a dose of 20 CFU/animal using *s.c.* route. Bacteriological evaluation was assessed by counting *Brucella* colonies in tissues, where data is expressed as log_10_ CFU/g and the index of infection of infected animals and compared to the control groups (the arithmetic mean ± standard error was given for each group; number of tissues from where *Brucella* was isolated for each animal). Statistical analysis for (A-C) was performed using a one-way ANOVA followed by Dunnett's multiple comparisons test and for (D) using two-way ANOVA, Tukey’s multiple comparisons test

According to the index of infection (Fig. 4d), a significant level of protection in comparison with the control group (level of infection: 100%) was achieved in all three tested doses and only after secondary boost vaccination (*P* < 0.0004, *P* < 0.02). The index of infection in the groups of animals immunized with a dose of vaccine 10^6^ EID_50_ and *B. melitensis* Rev.1 vaccine was comparable with each other and these groups differed significantly compared to the negative control group *P* = 0.003–0.001.

It should be noted that the values of the index of infection between the *B. melitensis* Rev.1 group and the experimental groups did not have significance (*P* > 0.05).

## Discussion

To our knowledge, this study is the first trial conducted in guinea pigs to evaluate the protective properties of a new candidate for vector vaccine against human brucellosis. This phase in vaccine trials is an important step in making an experimental vaccine a promising candidate for further human clinical trials to determine its effectiveness. In this study, double *i.n.* immunization with a vector vaccine based on influenza viral vectors expressing the immunodominant brucellosis proteins Omp16, L7/L12, Omp19 and Cu–Zn SOD at a dose of 10^6^ EID_50_ ensured protection against *B. melitensis* 16 M infection comparable to the effect of commercial *B. melitensis* Rev.1 vaccine.

The choice of guinea pigs as model animals for evaluation of body gain changes and vaccine candidate protection was determined by their natural resistance to influenza infection in comparison with laboratory mice. In this case, the use of a more resistant model animal seemed to be a key condition in the study of protection, since, in the long run, the vaccine is designed for humans.

The previous success of using IVV in the development of the anti-brucellosis vaccine Flu-BA for cattle [12], which is now at the stage of commercialization in Kazakhstan, served as the basis for this study. The idea of developing an anti-brucellosis human vaccine is that the high efficacy of the vaccine is achieved in cattle which naturally resistant to influenza infection (i.e., as a non-replicable viral vector), and, in our opinion, should be even more pronounced in humans. This assumption is based on the fact that humans are a natural host for the influenza virus (Influenza A), including the influenza viral vectors we use.

It should be noted that we used an influenza viral vector (IVV) of the H5N1 subtype, because there is no immune background to this type of pathogen [22] in the human population and IVV of the H5N1 subtype has a greater potential as a vaccine vector.

We began the process of creating an effective anti-brucellosis vector vaccine for humans with the formation of requirements for the developed product, production technology and methods of its application and testing in healthcare practice. To this end, we have accumulated the existing experience in the development of vector vaccines for public health, have chosen the most generally accepted requirements for such vaccines and their manufacturing technologies, the method and frequency of their use and have developed criteria for assessing the effectiveness and safety of vaccine candidates.

An analysis of the compliance of the developed vaccine with the above requirements (which are more general in nature than specific) showed that the viral vectors we selected, as well as the method for preparing and using the vaccine, correspond to them. In particular, we use non-pathogenic influenza viral vectors, the general safety of which has been confirmed by studies on guinea pigs with various ways of administering and dose of immunization.

In order to obtain influenza viral vectors (IVV), we used A/Puerto Rico/8/34 (H1N1) with a length-modified NS1-80 gene encoding 80 amino acids in the N-terminal region of the protein as the initial strain. The surface genes of hemagglutinin (HA) and neuraminidase (NA) were taken from A/chicken/Astana/6/05 strains (H5N1, with the HA cleavage site preliminary removed). The safety or attenuation of IVV is ensured by the truncated NS1 protein (interferon antagonist), which results in their limited replicative capabilities (they make one cycle of reproduction in the cell and do not leave it) [14]. It is known that the degree of IVV attenuation is directly dependent on the length of the NS1 protein [23]. We have an IVV with NS1 length in 80 amino acid. NS1-124 was used to create a veterinary brucellosis vaccine as it for use in cattle a more aggressive IVV was required. As for humans, far preferable may be IVV with NS1-80. With IVV subtype H5N1 (a pathogenic variety of influenza virus), the attenuation was additionally achieved by removing the proteolytic cleavage site in the HA protein, that is, double attenuation was performed. During repeated re-inoculation in chick embryos, IVV retained all their basic biological properties, including signs of attenuation, and did not lose the brucellosis insertion segment [14], which indicates their genetic stability. In addition, the influenza viral vectors we use are RNA-containing viruses that are limited to cytoplasmic replication, thus eliminating the risk of integration and long-term persistence.

The next important phase of our research was devoted to the study of the general safety control of the vaccine candidate at the early stage with different ways of administration and dose of use in guinea pigs. The vaccine has been found to be safe for guinea pigs when administered *c.*, *i.n.* and *s.l.* The experimental animals did not show death or signs of any disease; by the end of observation (on day 14 after the prime-boost vaccinations), the body weight gain in guinea pigs was observed both after prime and after boost immunization. At the same time, the increase in body weight of guinea pigs in the experimental groups was comparable to the control group of animals that were injected with PBS. As a result of this work, the vaccine was recognized as a safe drug and was used in the future to assess its protectiveness depending on the immunization schedule.

Further assessment of the effectiveness of the vaccine with different routes of administration to mucosal areas was determined using *c.*, *i.n.* and *s.l.* methods of vaccine immunization in prime-boost mode. Since the influenza virus has a tropism for mucosal surfaces, it was assumed that the optimal way to administer a vaccine based on an influenza viral vector would be one of the tested mucosal routes. Since *Brucella* should be considered as a mucosal pathogen penetrating mucous surfaces, the “gates” of infection are the mucosal surfaces of the nose or mouth. Consequently, mucosal vaccination is capable to generate protective responses against pathogens at the site of the infection “gate” [24]. Our bacteriological study demonstrated that significant protection of guinea pigs after challenging with virulent strain of *B. melitensis* 16 M infection was achieved through *i.n.* administration of the vaccine in comparison with other methods of application.

The next important step in our study was devoted to the choice of the vaccination dose and, at the same time, the frequency of vaccination, where the study of the protection and immunogenicity of the vaccine candidate was evaluated in animals by the ability to retain bacteria in organs and lymph nodes after animal infection with standard methods. Another distinctive feature of our studies was that the vaccine protection was assessed not only by the *Brucella* culture isolation from the tissues of vaccinated and unvaccinated animals, but also by such aspects as vaccination efficiency and infection index. It is believed that these indicators jointly provide a more complete and objective characterization of the vaccine protection. The new vaccine induced significant protection in response to *B. melitensis* 16 M infection within a range of 60–80% when administered *i.n.* in a double vaccination mode for all tested doses, and it was not inferior in efficiency to *B. melitensis* Rev.1, which is currently used in veterinary practice as the most immunogenic brucellosis vaccine. The level of protection of the *B. melitensis* Rev.1 vaccine obtained in our studies corresponds to the science literature data [25]. At the same time, it was found that the new vaccine candidate does not possess protection after primary vaccination, regardless of the dose. When choosing an immunizing dose of the vaccine, it is recommended to use a vaccination dose of 10^6^ EID_50_, since the protection at the 10^6^ EID_50_ dose (80% efficiency) was higher than 10^5^ EID_50_ (60% efficiency) and similar to 10^7^ EID_50_ (80% efficiency). The choice of an immunizing dose of 10^6^ EID_50_ is determined by the reduction of possible adverse effect of vaccination and the cost of the production process of the vaccine. The vaccine is targeted at a specific risk group—laboratory scientists working with the pathogen, veterinarians, slaughterhouse workers and people involved in animal care industry. The next step in the further vaccine development will be devoted to the preclinical studies where will be evaluated the safety, immunogenicity and protectiveness of a new human vaccine candidate against brucellosis.

## Conclusions

The results of the study in guinea pig models show that the recombinant vector anti-brucellosis vaccine candidate is a safe product with wide dose of application and mucosal immunization techniques and its protection properties in prime-boost immunization mode are comparable to that of the commercial *B. melitensis* Rev.1 vaccine against *B. melitensis* 16 M infection. We conclude the immunization schedule for our new candidate vaccine against human brucellosis—tetravalent vaccine formulation based on recombinant influenza A virus subtype H5N1 expressing *Brucella* Omp16, L7/L12, Omp19 and SOD in prime-boost intranasal immunization mode at immunization dose of 10^6^ EID_50_. We could further use this recombinant vaccine vector for pre-clinical and clinical trials in humans.

## Data Availability

All data related to the present study are available in the manuscript.

## Abbreviations

EID: Embryo infectious dose
CFU: Colony forming units
rIVV: Recombinant influenza viral vector
Omp: Outer membrane protein
PBS: Phosphate-buffered saline
HA: Hemagglutinin
NA: Neuraminidase
PB2: PB1 and PA, Influenza A virus RNA polymerase subunits
NP: Nucleoprotein
M: Matrix
NS1: Non-structural protein 1
NEP: Nuclear export protein
